# IFNγ Modulates the Immunopeptidome of Triple Negative Breast Cancer Cells by Enhancing and Diversifying Antigen Processing and Presentation

**DOI:** 10.3389/fimmu.2021.645770

**Published:** 2021-04-22

**Authors:** Gabriel Goncalves, Kerry A. Mullan, Divya Duscharla, Rochelle Ayala, Nathan P. Croft, Pouya Faridi, Anthony W. Purcell

**Affiliations:** Department of Biochemistry and Molecular Biology, Monash Biomedicine Discovery Institute, Monash University, Clayton, VIC, Australia

**Keywords:** mass spectrometry, immunopeptidomics, triple negative breast cancer, cytokine stimulation, human leukocyte antigen, transcriptomics, proteomics

## Abstract

Peptide vaccination remains a viable approach to induce T-cell mediated killing of tumors. To identify potential T-cell targets for Triple-Negative Breast Cancer (TNBC) vaccination, we examined the effect of the pro-inflammatory cytokine interferon-γ (IFNγ) on the transcriptome, proteome, and immunopeptidome of the TNBC cell line MDA-MB-231. Using high resolution mass spectrometry, we identified a total of 84,131 peptides from 9,647 source proteins presented by human leukocyte antigen (HLA)-I and HLA-II alleles. Treatment with IFNγ resulted in a remarkable remolding of the immunopeptidome, with only a 34% overlap between untreated and treated cells across the HLA-I immunopeptidome, and expression of HLA-II only detected on treated cells. IFNγ increased the overall number, diversity, and abundance of peptides contained within the immunopeptidome, as well increasing the coverage of individual source antigens. The suite of peptides displayed under conditions of IFNγ treatment included many known tumor associated antigens, with the HLA-II repertoire sampling 17 breast cancer associated antigens absent from those sampled by HLA-I molecules. Quantitative analysis of the transcriptome (10,248 transcripts) and proteome (6,783 proteins) of these cells revealed 229 common proteins and transcripts that were differentially expressed. Most of these represented downstream targets of IFNγ signaling including components of the antigen processing machinery such as tapasin and HLA molecules. However, these changes in protein expression did not explain the dramatic modulation of the immunopeptidome following IFNγ treatment. These results demonstrate the high degree of plasticity in the immunopeptidome of TNBC cells following cytokine stimulation and provide evidence that under pro-inflammatory conditions a greater variety of potential HLA-I and HLA-II vaccine targets are unveiled to the immune system. This has important implications for the development of personalized cancer vaccination strategies.

## Introduction

Breast cancer is the most commonly diagnosed cancer and contributes to the second largest mortality rate in women worldwide (1). Triple Negative Breast cancer (TNBC) is a highly heterogeneous disease characterized by malignant and aggressive tumor growth within the breast ducts (2). TNBC is characterized by the negative expression of three extracellular receptors: the estrogen receptor (ER), the progesterone receptor (PR), and the human epidermal growth factor receptor 2 (HER2) (3). Consequentially, this has led to many therapeutic challenges in pursuing avenues for treatment, as TNBC is not subject to targeted therapies that are exploited in traditional hormone-dependent breast cancer subtypes (4). As well-defined molecular targets for TNBC await identification, chemotherapy remains the primary means for treatment (5). As such, novel therapies are being explored, particularly those drawing upon the recent advancements in T cell immunotherapy.

Harnessing the power of the adaptive immune response is becoming an area of much interest and promise. Since the discovery of the first T cell-defined tumor antigen (6), a plethora of studies have led to the identification and direct assessment of the cell surface HLA-peptide repertoire (the field of immunopeptidomics) in different cancers, autoimmunity related conditions, and viral infections (7–14). These HLA-peptides can derive from the HLA-I pathway *via* cytosolic proteasomal degradation of proteins and transport of resultant peptides to the endoplasmic reticulum by the transporter associated with antigen processing (TAP) (15). These peptides (typically 8–12 amino acids in length) are loaded onto nascent HLA-I molecules based on their suitability to bind to the individual’s HLA allotypes and transported to the cell surface for CD8+ T cell recognition. In contrast, the HLA-II pathway relies on lysosomal degradation of proteins by professional antigen presenting cells (APCs), with peptide loading occurring in the late endosome (with peptides 13–17 amino acids in length) (16) and transport of the HLA-II-peptide complexes to the cell surface for recognition by CD4+ T cells.

The molecular apparatus governing cell surface peptide display by HLA molecules is referred to as the antigen processing and presentation machinery (APPM), and both the HLA-I and HLA-II pathways have been shown to be strongly influenced by the tumor microenvironment including the presence of the pro-inflammatory cytokine interferon γ (IFNγ) (17, 18). IFNγ has the capacity to augment the presentation of HLA-I molecules (particularly the HLA class I B allomorphs) on the cell surface, as well as by altering the peptide repertoire through inducing the formation of the immunoproteasome (8, 19–23). This specialized form of the proteasome possesses altered cleavage specificities, and has been proposed to remold the peptide repertoire allowing for presentation of a more diverse set of peptide antigens (8, 19, 20, 24). Furthermore, cytokine driven changes have the ability to induce HLA-II expression on non-professional APC cells by activating the MHC class II transactivator (CIITA) (25). This phenotype can be mimicked in cell culture experiments following IFNγ treatment allowing for the elucidation of HLA-II targets on cancerous cells that would otherwise be missed. Despite these known effects of IFNγ, the mechanism of how cytokine derived alterations to the peptide repertoire occur has been a source of conjecture, with the individual roles of the transcriptome, source protein expression, and changes within the APPM yet to be conclusively determined. This is compounded by the poor correlations often observed between the proteome and the transcriptome of cells (26, 27). Further, the general lack of immunopeptidomic studies with corresponding transcript and source protein measurements has led to difficulties elucidating the key determinants of HLA peptide presentation.

Here we have investigated the composition of the TNBC cell line MDA-MB-231 transcriptome, proteome, and immunopeptidome and quantified changes observed in each ‘Omics data set under conditions of IFNγ treatment. We achieved substantial depth at the transcript, protein, and HLA-peptide level and show that IFNγ not only increases antigen presentation but also unveils a suite of novel peptides to the immune system, many of which derive from known cancer antigens. This remolding of the peptidome was not strongly correlated with changes observed at transcript and protein level. These data highlight the importance of directly measuring the immunopeptidome of cancer cells since antigen processing and presentation cannot be inferred from the more readily measured transcriptome and proteome. Therefore, studies relying on these assumptions, risk excluding and misidentifying bona fide and potential therapeutic targets for cancer vaccination.

## Materials and Methods

### Cell Culture

MDA-MB-231 cells were cultured in DMEM supplemented with 10% fetal bovine serum, 1% Penicillin/streptomycin and L-glutamine (2 mM) (Gibco) at 37°C with 5% CO2. Cells were titrated at 50,100 and 200 IU of lyophilized human IFNγ (Miltenyi Biotec #130-096-484) for 48 h before being examined at a time course across 16, 24, 48-h intervals. IFNγ treated cells were then treated with 50 IU of lyophilized human IFNγ (Miltenyi Biotec #130-096-484) for 48 h for all subsequent experiments with all cells being cultured from a single seed flask for comparative analysis.

### HLA Typing of MDA-MB-231 Cells

Total DNA was extracted from 5 × 10^6^ cells by the Qiagen DNA extraction for spin-column or 96-well purification of total DNA from animal blood and tissues and from cells, yeast, bacteria, or viruses (cat # 69504) kit as per manufacturer’s protocol. Samples were sent to the Victorian Transplantation and Immunogenetic Service (VTIS) for high resolution HLA typing.

**Table:** 

MDA-MB-231 TNBC cell line HLA haplotype.			
Class I alleles	A allele	B allele	C allele
	02:01, 02:17	40:02, 41:01	02:02, 17:01
Class II alleles	DR allele	DP allele	DQ allele
	07:01, 13:05	02:01, 17:01	02:02, 03:01

### Flow Cytometry

HLA complexes were stained with a pan HLA-I antibody w6/32 conjugated to PE and a pan HLA-II antibody RM5.112 also conjugated to PE. All flow cytometric assays were acquired on a BD LSRFortessaTM X20C cell analyzer [LSRII (Becton Dickinson Biosciences)] located in the FlowCore facility at Monash University. Data analysis was performed on Tree Star FlowJo^®^ software (Becton, Dickinson & Co).

### RNA Sequencing

Total RNA was extracted from 1 × 10^5^ MDA-MB-231 cells in three biological replicates of IFNγ treated (50 IU for 48 h) and untreated conditions using RNeasy mini kit (Qiagen) according to the manufacturer’s instructions. Cells were cultured from an initial seed flask before being split into six flasks for comparative analysis of each replicate. Sequencing was performed by BGI Genomics (Hong Kong) with 100 base pair end reads to a depth of 30 million reads.

The raw RNA-sequencing files were aligned using the Monash Bioinformatics Platform RNAsik pipeline (28). The RNA-seq files were aligned to the GRCh37 version of the genome and the corresponding annotation files (gtf).

### Processing of the Raw Count File

The raw files were imported into R studio (R version 3.5.3: “Great Truth”) (29). The package “EdgeR” (30) was used to create the generalized linear models to determine which genes were differentially expressed. Additionally packages used for the analysis included: “ggplot2” (31), “dplyr” (32), “ggrepel” (33), “tidyr” (34). A false discovery rate (FDR) <0.05 was considered statistically significant.

### Proteomics

The total proteome content of MDA-MB-231 cells was extracted in three biological replicates of IFNγ treated (50 IU for 48 h) and untreated conditions. These cells were cultured from the identical seed flask used in transcriptome analysis before been split into an additional six flasks and harvested. Harvested cells were snap frozen using liquid nitrogen on the same day for proteomics and transcriptomics studies. These pelleted cells were lysed (5 × 10^6^ cells) with 1% (w/v) sodium deoxycholate SDC lysis buffer in Tris pH 8.1. Lysate was then boiled at 95°C for 5 min and sonicated. Then 400 µg of protein was taken, and this was then denatured and alkylated with the addition of 10 mM Tris(2-carboxyethyl) phosphine (TCEP) and 40 mM chloroacetamide (CAA) at 95°C for 5 min. This was then allowed to cool to room temperature and the pH was adjusted to 8 before trypsin was added at a ratio of 1:30 [trypsin:protein (w/w)] and incubated at 37°C with shaking at 180 rpm overnight. The trypsin digested lysate was then fractionated into 18 fractions by C18 RP-HPLC. These fractions were then pooled into six fractions and analyzed on mass spectrometry for data dependent acquisition (35).

### Immunopeptidomics Sample Preparation

HLA-I and -II peptides were eluted from three biological replicates of 5 × 10^8^ MDA-MB-231 cells prior to or after treatment with IFNγ (i.e. n = 6) as described (36). Briefly, cells were lysed in 0.5% IGEPAL, 50 mM Tris-HCl pH 8.0, 150 mM NaCl supplemented with protease inhibitors (Complete Protease Inhibitor Cocktail Tablet; Roche Molecular Biochemicals) for 45 min at 4°C. Lysates underwent ultracentrifugation at 40,000 g and HLA-I and -II complexes were purified using a pan HLA-I antibody (w6/32) whilst HLA-II complexes were purified with the allele-specific antibodies of LB3.1 (HLA-DR), B721 (HLA-DP), and SPV-L3 (HLA DQ). The HLA peptide bound molecules were then eluted from the affinity column with five column volumes of 10% acetic acid. The eluted HLA-I or HLA-II peptide complexes were loaded onto a 4.6 mm internal diameter × 50 mm monolithic C18 RP-HPLC column (Chromolith Speed Rod; Merck) at a flow rate of 1 ml/min using an EttanLC HPLC system (GE Healthcare) with buffer A [0.1% trifluoroacetic acid (TFA)] and peptides separated by increasing concentrations of buffer B (80% ACN/0.1% TFA) as described in (36). Fractionated peptides were then concatenated into 10 pools and analyzed with an Orbitrap Fusion™ Tribrid™ mass spectrometer (ThermoFisher Scientific).

### LC-MS/MS Data Acquisition

For identification of peptides for both proteomics and immunopeptidomics we used Data dependent acquisition (DDA) approach. Using a Dionex UltiMate 3000 RSLCnano system equipped with a Dionex UltiMate 3000 RS autosampler, the samples were loaded *via* an Acclaim PepMap 100 trap column (100 µm × 2 cm, nanoViper, C18, 5 µm, 100Å; Thermo Scientific) onto an Acclaim PepMap RSLC analytical column (75 µm × 50 cm, nanoViper, C18, 2 µm, 100Å; Thermo Scientific). The peptides were separated by increasing concentrations of 80% ACN/0.1% FA at a flow of 250 nl/min for 65 min and analyzed with a QExactive Plus mass spectrometer (Thermo Scientific). In each cycle, a full ms1 scan (resolution: 70.000; AGC target: 3e6; maximum IT: 120 ms; scan range: 375–1800 m/z) preceded up to 12 subsequent ms2 scans (resolution: 17.500; AGC target: 1e5; maximum IT: 120 ms; isolation window: 1.8 m/z; scan range: 200–2,000 m/z; NCE: 27).

For quantitative proteomics we used data independent acquisition (DIA) approach the identical LC-MS/MS instrument setup above was used. Twenty-five sequential DIA windows with an isolation width of 24 m/z between 375 and 975 m/z were acquired. A 158-min gradient of increasing concentrations of 80% ACN/0.1% FA was used to separate the peptides for DIA acquisition.

### Proteomics Identification and Quantification

The Acquired DDA.raw files were searched against the human proteome (Uniprot v_05102017) using Spectronaut Pulsar software to obtain peptide sequence information and generate a spectral library for DIA analysis. The following search parameters and settings have been used: (i) trypsin (full specificity after Arginine and Lysine) was selected as protease and up to one missed cleavage was permitted (37); mass tolerances were set to 10 ppm for precursor and 0.02 Da for fragment masses; (iii) carbamidomethylation of C was selected as fixed and oxidation of methionine residues was selected as variable modification. Only peptides identified at a false discovery rate (FDR) of 1% based on the added decoy database were considered for spectral library generation. Spectronaut 10 (Orion; Biognosys) was used to create the corresponding spectral library and search all DIA raw files. For data analysis, we used Spectronaut 10 default protein‐centric parameters (BGS Factory Settings). Data matrices containing the quantitative values were obtained from “QValue Sparse” scores.

### HLA Peptide Identification

MS/MS spectra were analyzed *via* PEAKS studio X software. MS files were imported into PEAKS X with the following settings: parent mass error tolerance was set at 10 parts per million with a fragmentation mass error of 0.02 Da. An initial *de novo* search of all MS/MS spectra against peptide sequences was performed followed by a specific search against the human proteome database with oxidation of methionine selected as a variable PTM. A false discovery rate (FDR) of 1% was implemented and all resulting peptides were exported.

### Clustering and Binding Prediction of HLA Peptides

The peptide data was extracted and underwent further data analysis using Gibbs clustering to validate the biochemical properties of these peptides (38). Furthermore, peptides were allocated as binders or non-binders using NetMHCpan. This software predicts HLA-peptide binding using artificial neural networks, here we implemented the default cut-off of 2 as a binder peptide (39).

## Results

### IFNγ Diversifies the Immunopeptidome of the TNBC MDA-MB-231 Cell Line

**Figure 1:**
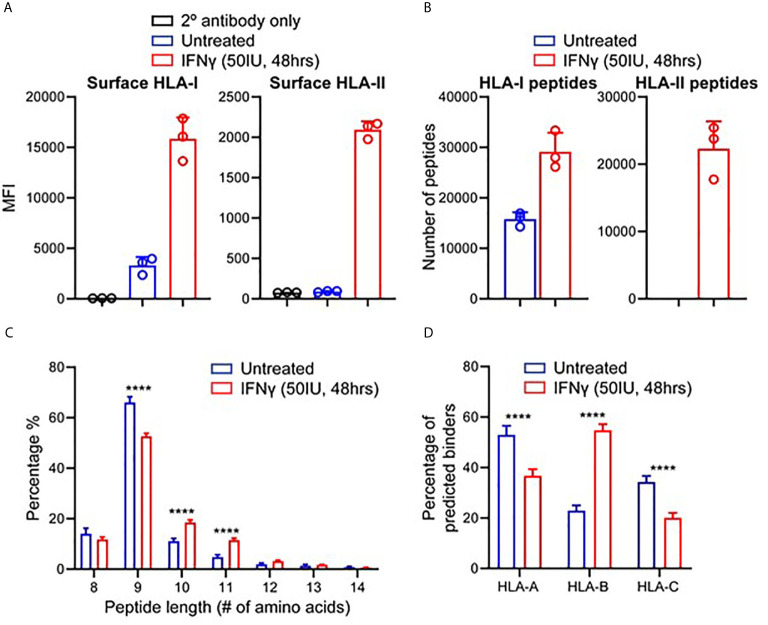
IFNγ modulates HLA expression and the immunopeptidome of MDA-MB-231 cells. **(A)** Flow cytometry staining of HLA class I and class II expression following IFNγ stimulation. Median fluorescence intensity (MFI) is shown across three biological replicates (mean ± SD). **(B)** Immunopeptidome profiling of MDA-MB-231 cells in the presence or absence of IFNγ, showing number of peptides for HLA-I and HLA-II. **(C)** Length analysis of unique HLA-I peptides derived from MDA-MB-231 cells with and without IFNγ treatment. **(D)** NetMHCpan 4.0 HLA-A, -B, and -C binding prediction of each unique 8–12 mer peptide from IFNγ treated and untreated replicates. Statistical analysis was performed by using a two-way ANOVA and significance is denoted by ****(P < 0.001). All data are from three biological replicates per condition.

To study the effect of IFNγ on the immunopeptidome of TNBC, we utilized MDA-MB-231, a metastatic breast adenocarcinoma cell line derived *via* pleural effusion (40). Testing IFNγ concentrations at multiple timepoints, we determined that 50 IU for 48 h led to the greatest increase in HLA-I expression (average of 4.8-fold) as well as inducing HLA-II expression (**Figure 1A** and **Supplementary Figure 1**) (P < 0.0001). These conditions were therefore used for all subsequent experiments.

Next, we interrogated the immunopeptidome of MDA-MB-231 cells in the presence or absence of IFNγ, *via* immunoprecipitation of total HLA-I or HLA-II and identification of eluted peptides by mass spectrometry (36). Across three biological replicates of IFNγ treated and untreated MDA-MB-231 cells, a total of 51,779 HLA-I and 34,641 HLA-II peptides were identified. An average of 15,785 HLA-I peptides were presented on untreated samples, increasing to 29,165 (1.85-fold p < 0.004) on IFNγ treated samples, whilst an average of 22,316 HLA-II peptides were observed across the IFNγ treated cells (**Figure 1B**).

To attain a more detailed insight into this modulation, we listed and categorized all HLA-I peptides identified in untreated and IFNγ treated samples and compared each of these against replicates from the opposing condition, allowing categorization of common peptides and those unique to each condition (**Supplementary Figures 2A–F**). Overall, the HLA-I immunopeptidome reflects a total unique repertoire of 27,532 peptides (53.2%) present only following IFNγ treatment. Conversely, 4,850 peptides (9%) were uniquely present on untreated samples whilst 19,397 (~37.5%) were shared across both HLA-I conditions (**Supplementary Figure 2G**). Analysis of the unique peptides showed that, as expected (and as observed in the common set), HLA-I peptides displayed a marked preference for 9 mers (**Figure 1C**). However, IFNγ stimulation led to a statistically significant increase (p < 0.0001 2way ANOVA for multiple comparisons) in the presentation of 10 and 11 mers (**Figure 1C**). As anticipated HLA-II peptides only detected under conditions of IFNγ treatment (**Supplementary Figure 3A**), and these peptides displayed characteristic length distributions predominantly (~65%) between 14 and 18 amino acids in length (**Supplementary Figure 3B**).

We next assessed the HLA allotype binding predictions of the peptides specific to treated or untreated cells. Filtering on 8–12 mer peptides unique to untreated or IFNγ treated, we analyzed these peptides according to their HLA allotype predicted binding affinities using NetMHCpan 4.0 (**Figure 1D** and **Supplementary Figure 3C**); 80.8% (1,756) of peptides unique to untreated cells and 90.2% (12,492) of peptides unique to IFNγ treated cells were assigned as predicted binders to at least one of the alleles expressed by MDA-MB-231. However, IFNγ treatment also resulted in a significant (p < 0.0001) increase (~32%) in the number of predicted binders to the HLA-B allomorphs expressed by these cells (**Figure 1D**). Notably, the increased preference for 10 mers and 11 mers was more pronounced for peptides identified following IFNγ treatment and particularly for HLA-B (**Supplementary Figures 3D–G**). Further evidence of HLA-B enrichment following IFNγ treatment was observed by performing Gibbs cluster analysis. This software clusters peptides based on distinct sequence features (motifs), and this revealed a higher proportion of 9 mer peptides were clustered to the motif of HLA:B rather to the other alleles reported for this cell line (**Supplementary Figures 4A, B**).

### IFNγ Treatment Alters the Source Protein Landscape of MDA-MB-231 Cells

**Figure 2:**
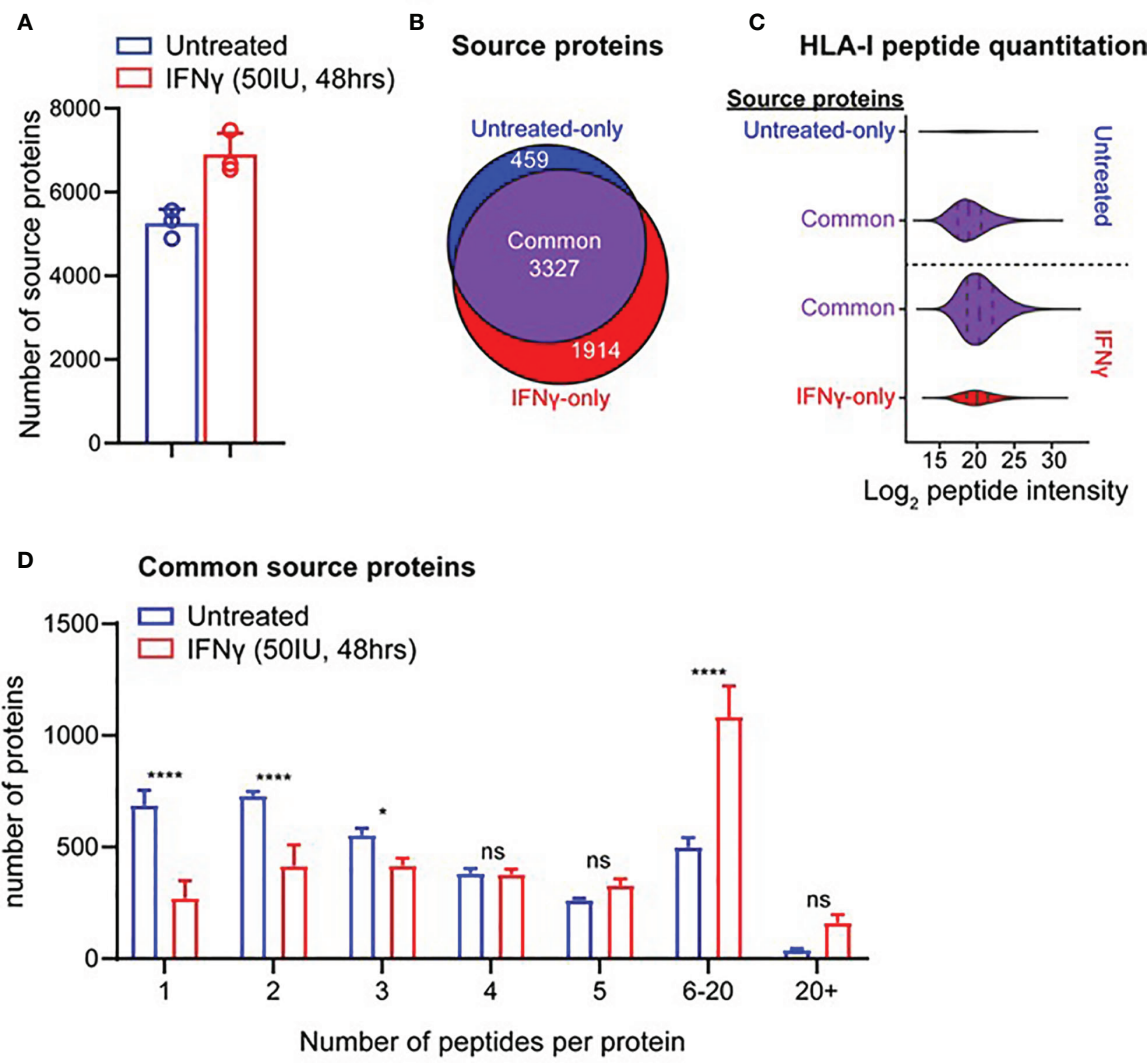
IFNγ causes considerable remolding of the source protein immunopeptidome landscape of TNBC cells. **(A)** Source proteins of HLA-I peptides identified across three biological replicates of IFNγ treated (mean of 6,897 proteins) and untreated samples (mean of 5,256 proteins). **(B)** Comparative analysis of source protein overlap between untreated and IFNγ treated cells. **(C)** HLA-I source peptide log_2_ intensity calculated from label free quantification from common and unique HLA-I source proteins. **(D)** Histogram of number of HLA-I peptides per source protein under untreated or IFNγ treated conditions. All data are from three biological replicates with mean ± SD and statistical analysis was performed by using a two-way ANOVA and significance is highlighted by ****(p < 0.001). * P < 0.1, ns, not significant.

To assess how IFNγ altered the presentation of different antigens in the HLA-I repertoire, we examined the source protein origin of identified peptides (**Figures 2A, B**). Comparison of source protein coverage between IFNγ treated and untreated replicates revealed that the number of source proteins increased (1.3-fold) following treatment (**Figure 2A**). A comparative analysis revealed that across three biological replicates, 3,327 source proteins were shared across the two conditions. Whilst IFNγ treatment led to 1,914 source proteins being uniquely represented at the immunopeptidome level (**Figure 2B**). Conversely, 459 proteins were only represented by HLA-I peptides under the untreated conditions (**Figure 2B**).

To further examine the effect of IFNγ on the immunopeptidome of MDA-MB-231 cells at a source protein level, we examined the degree of peptide coverage and relative peptide abundance for all 3,327 shared HLA-I source proteins. Source proteins of peptides isolated from untreated cells contained a mean peptide coverage of 6.10%, whilst following IFNγ this increased significantly by 1.44-fold to a mean of 8.82% (p < 0.0001) (**Supplementary Figure 5A**). A similar effect was observed when comparing total peptide number across these antigens with a 1.68-fold increase following IFNγ treatment (mean of 4.074 for untreated *vs* 6.835 for IFNγ treated, p < 0.0001) (**Supplementary Figure 5B**). Label-free quantitation (LFQ) of the MS1 intensity of these peptides also showed a significant shift (~2.8-fold, p < 0.0001) following IFNγ treatment, whilst peptides derived from proteins unique to each condition had lower LFQ values for untreated, increasing ~1.87-fold upon exposure to IFNγ (**Figure 2C**).

Following the changes observed within the intensities of the common source proteins, we also determined whether the number of peptides derived from each source protein was altered following IFNγ treatment. As seen in **Figure 2D**, IFNγ treatment caused a significant decrease in the number of proteins that are represented by only one or two HLA-I peptides (p = 0.0001 and p = 0.0018 respectively), whilst there was a significant increase observed in the number of proteins that are represented by 6–20 (p < 0.0001) distinct peptides. Based on this increased coverage of source proteins observed within the immunopeptidome following IFNγ treatment, we next investigated the relevance of this observation for antigens that may be of clinical interest.

### IFNγ Increases the Presentation of HLA-Peptides Derived From Tumor Associated Antigens

**Figure 3:**
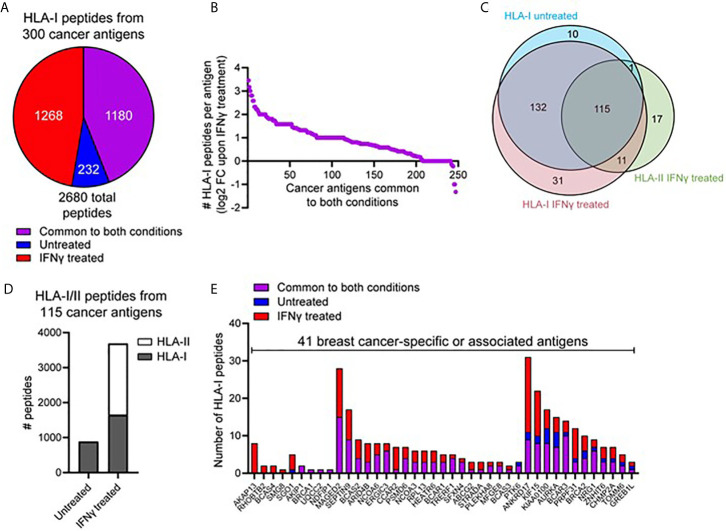
IFNγ leads to increased representation of cancer-associated antigens across the HLA-I and -II immunopeptidome. **(A)** Pie chart showing the total number of HLA-I peptides identified from 300 cancer-associated antigens (derived from the T antigen, CTdatabase, and breast cancer specific antigens from Uniprot), either unique to untreated, unique to IFNγ treated, or common to both conditions. **(B)** Log_2_ fold-change in the number of HLA-I peptides per protein detected following treatment with IFNγ. The 245 proteins subset are cancer-associated antigens represented across the HLA-I immunopeptidome under both conditions. **(C)** Overlap of cancer-associated source proteins across the HLA-I (untreated and IFNγ treated) and HLA-II (IFNγ treated) immunopeptidomes. **(D)** The total number of peptides from the 115 commonly identified cancer-associated antigens within the HLA-I -II immunopeptidomes, under untreated or IFNγ treated conditions. **(E)** A refined subset of specific or associated breast cancer antigens (41 in total), showing the overall distribution of HLA-I peptides identified across these source proteins. Proteins are ordered based on the conditions they were detected under.

We next interrogated the HLA-I immunopeptidome from MDA-MB-231 cells for peptides derived from 641 breast cancer-specific or associated antigens compiled from multiple databases (CTantigen, T-Antigen, and breast cancer-specific antigens from UniProt). Using this refined list of 598 cancerous antigens (**Supplemental File 1**), a total of 2,680 peptides derived from 300 antigens were identified across our HLA-I immunopeptidome data in at least one replicate at 1% FDR. The IFNγ-dependence for the presentation of these peptides is shown in **Figure 3A**. This highlights that although 1,180 peptides derived from cancer antigens are represented in the HLA-I immunopeptidome under both conditions, IFNγ treatment leads to the presentation of an additional 1,268 peptides derived from cancer antigens (**Figure 3A**). Furthermore, from the 245 HLA-I cancer-associated antigens common to both conditions, many were represented by more peptides following IFNγ treatment (**Figure 3B**) (A similar trend was observed across the total common source antigen pool, **Supplementary Figure 5C**).

We next assessed the overall representation of these cancer associated antigens across the HLA-I and -II immunopeptidomes under both conditions (**Figure 3C**). This analysis revealed that whilst many antigens had peptides presented by both HLA-I and HLA-II, 17 antigens were identified to be exclusive to the repertoire of HLA-II. Notable within these cancer antigens were the GTPase KRAS and the breast carcinoma associated antigen DF3. To perform a direct comparison between the repertoires and coverage of HLA-I and HLA-II in the context of these antigens and IFNγ treatment, we examined the 115 shared (**Figure 3C**) cancer associated antigens present in the HLA-I and HLA-II peptide datasets. From these 115 antigens, 885 peptides were presented by HLA-I under normal conditions whereas upon IFNγ treatment this increased ~4.2-fold to a total of 3,688 peptides from across both HLA-I (1,644 peptides) and HLA-II (2044 peptides) (**Figure 3D**).

The above dataset of 300 cancer-associated antigens presented by HLA-I was further refined to only include those described as associated or specific to breast cancer, resulting in 41 antigens in total. **Figure 3E** shows the number of HLA-I peptides derived from these proteins under each condition (classified as only detected in untreated, only detected in IFNγ-treated or common to both conditions). This exemplifies different trends such as the four-breast cancer associated antigens (A-kinase anchor protein 13, Rho-related BTB domain-containing protein 2, Breast carcinoma-amplified sequence 4, and Protein SMG8) only present within IFNγ treated cells. Furthermore, this analysis highlights the capacity of IFNγ to enrich peptides associated with these cancer antigens, with a large proportion of these antigens only detected in IFNγ dependent immunopeptidome (**Supplementary Figure 6A** denotes the total cancer antigen pool, which further exemplifies this trend). Examples of specific antigens of interest that highlight the capacity of IFNγ to augment the presentation of potential breast cancer-associated candidates include NY-BR-16, which increased from 11 peptides to 30 following IFNγ treatment, and breast cancer-associated antigen BRCAA1, which increased from three peptides to eight following IFNγ treatment. Furthermore, from the 2,680 total HLA-I peptides derived from these cancer antigens a total of 636 were previously unreported in the literature, further highlighting the depth and coverage of this repertoire.

### Integrated Omics Reveal Increased APPM Modulation Following IFNγ Treatment

**Figure 4:**
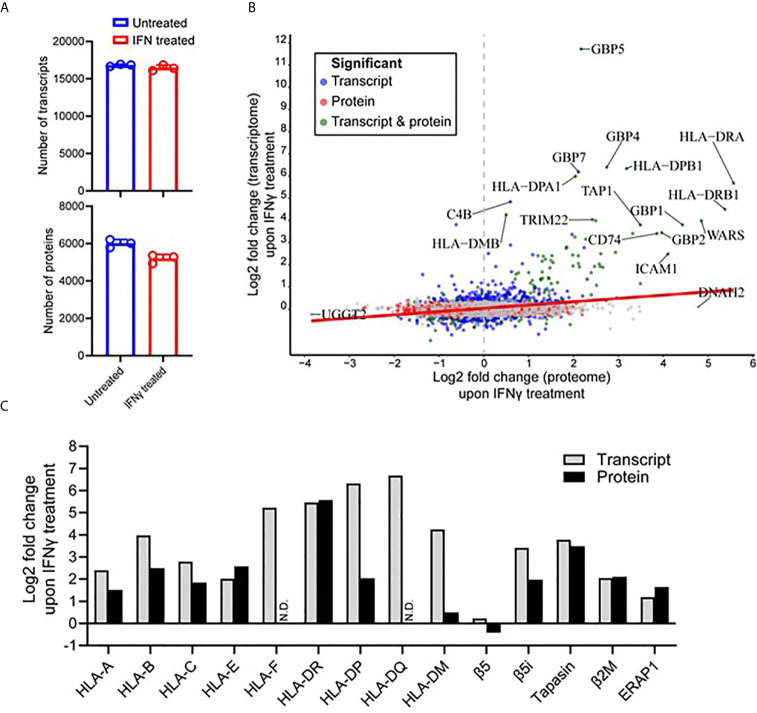
IFNγ treatment predominantly induces components of the antigen processing and presentation machinery of MDA-MB-231 cells. **(A)** The number of transcripts (upper) and proteins (lower) identified from untreated and IFNγ treated cells. Data are from three biological replicates. *p = 0112 by unpaired t-test. **(B)** Correlation between the Log_2_ fold change (IFNγ treated compared to untreated) transcripts and proteins. Significant fold changes at the protein (red), transcript (blue), or protein and transcript (green) level are indicated, with the gene symbols of those with the highest fold-changes indicated. Person correlation coefficient of 0.275, p value <0.0001. **(C)** The log_2_ fold change of transcriptome and proteome changes within key components of the antigen processing and presentation machinery following IFNγ treatment.

To attain a further insight into the IFNγ-induced changes within the immunopeptidome, we examined the cellular transcriptome and proteome of the MDA-MB-231 cells to ascertain whether these changes were mirrored in these compartments. As Total RNA and cellular proteins were extracted from the identical seed flask of MDA-MB-231 cells and grown under the same conditions used for the immunopeptidome studies, we justify this rationale to perform integrative omics. Following RNA isolation and sequencing, an average of 16,815 and 16,452 transcripts for untreated and IFNγ treated conditions, respectively, were identified across the biological replicates (**Figure 4A**). From this analysis, 16,875 total transcripts were identified of which 1,094 were significantly up-regulated (FDR < 0.05) and 914 were significantly down-regulated (FDR < 0.05) after IFNγ treatment (**Supplementary Figure 7A**). The significantly up-regulated transcripts displayed a strong molecular signature of IFNγ inducible targets and highlight a profound effect on the APPM (**Supplementary Figure 7B**).

At the protein level, initial mass spectrometric analysis led to the identification of a mean of 5,191 proteins detected under IFNγ treated conditions compared to 6,004 proteins under untreated conditions (**Figure 4A**) (p = 0.0112). A more in-depth data-independent acquisition (DIA)-based quantitative mass spectrometry analysis revealed 463 proteins were found to be significantly up-regulated (Q-value <0.05) upon IFNγ treatment, whereas 812 proteins were significantly down-regulated (Q-value <0.05) (**Supplementary Figure 7C**). Similar to what was observed at the transcript level, a variety of downstream interferon mediated proteins and components of the APPM were enriched within the upregulated subset (**Supplementary Figure 7B**). The transcriptome and proteome overlap (**Supplementary Figure 7D**) allowed for comparative analysis of differential expression amongst shared genes and proteins, revealing a significant but weak correlation of 0.275 (p value <0.0001) (**Figure 4B**).

Following a clear indication that components and associated proteins of the APPM were highly susceptible to IFNγ treatment, we decided to further interrogate these changes (**Figure 4C**). Interestingly, within the HLA-I allotypes, non-classical HLA-I contained the largest increase following IFNγ treatment in the transcriptome (HLA-F was upregulated 37.5-fold) and the proteome (HLA-E was upregulated 5.96-fold). This was followed by HLA-B in both the transcriptome and the proteome, being up-regulated 15.74-fold and 5.64-fold, respectively. HLA-II allotypes were heavily modulated following IFNγ treatment at both the protein and transcript level. For example, HLA-DQ showed the highest increase within the transcriptome followed by HLA-DP and then HLA-DR. Although, this overall effect was maintained at the proteome level, the order was altered with HLA-DR showing the highest degree of modulation followed by HLA-DP. No HLA-F or HLA-DQ was detected *via* mass spectrometry (**Figure 4C**).

### Multi-omic Comparison of Proteome and Transcriptome Expression and Its Relation to the Immunopeptidome

**Figure 5:**
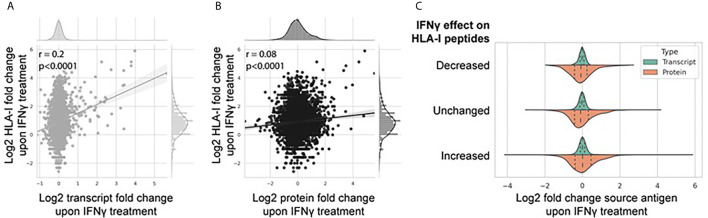
IFNγ-mediated modulation of the immunopeptidome is only weakly reflected by changes in the transcriptome and proteome. Correlation regression plot between the log_2_ fold change of the number of HLA-I peptides per protein with the associated log_2_ fold change of source transcript **(A)** and source protein **(B)** following IFNγ treatment. **(C)** Distribution of source transcript and protein log_2_ fold change, binned into categories of HLA-I peptide per protein presentation following IFNγ treatment [decreased (<0.5-fold); unchanged (0.5-fold to 1.5-fold); increased (>1.5-fold)].

The breadth and depth of this omics data allowed a systematic analysis between transcriptome and proteome abundance and resulting HLA-I derived peptides. To do this, we assessed the extent to which IFNγ-mediated differential expression at the transcript and protein level affected modulation of the immunopeptidome (**Figures 5A, B**). **Figure 5A** shows a weak but significant correlation (Pearson correlation coefficient 0.2, p < 0.0001) of IFNγ-induced changes at the transcript and HLA-I immunopeptidome level. A similar but weaker trend was observed at the protein level (**Figure 5B**; Pearson correlation coefficient 0.08, p < 0.0001). To assess this further, we categorized the change in the IFNγ-modulated immunopeptidome into three groups based on the fold change in the number of peptides per protein [*decreased* (fold change <0.5), *unchanged* (fold change 0.5–1.5), or *increased* (fold change >1.5)] and visualized the distribution of corresponding source transcript and protein expression across each category (**Figure 5C**). These data further suggest that the IFNγ induced modulation of the immunopeptidome is poorly reflected by corresponding changes across either the transcriptome or proteome.

Protein half-life has been suggested by previous studies to be an important determinant of HLA presentation, as proteins with high turnover rates are more susceptible to be presented *via* HLA molecules. To examine this we used the protein half-life data from the Lamond group (41) to determine the effect of protein half-life on our HLA peptide data. We examined our IFNγ treated and untreated datasets and could not find a significant correlation between proteins with shorter half-lives and an increased presentation on the immunopeptidome {Pearson correlation coefficient value of −0.004 [p-value of 0.8 (IFNγ untreated)] and −0.02 [p-value of 0.15 (IFNγ treated)]} (**Supplementary Figures 8A, B**). In order to further determine the impact of protein stability and its effect on HLA peptide presentation we filtered the Lamond dataset and used the top 100 most rapidly degrading proteins (RDP) and the top 100 most stable proteins to determine if a correlation could be observed between their respective protein half-life and the number of peptides presented on the immunopeptidome. Analysis between the top 100 half-lives of RDP and the number of HLA-I peptides by a Pearson correlation test, determined a correlation coefficient of 0.131 and 0.072 for untreated and IFNγ treated with non-significant p-values of 0.19 and 0.47 respectively (**Supplementary Figures 8C, D**). Analysis of the top 100 most stable protein half-lives with a Pearson correlation test yielding a correlation coefficient of 0.025 and 0.019 with non-significant p values of 0.81 and 0.85 respectively for untreated and IFNγ treated samples (**Supplementary Figures 8E, F**).

## Discussion

The tumor microenvironment (TME) is a pivotal component of tumor development and progression and is a key variable often overlooked when comparing cell culture experiments to patient derived cancerous material (8). The pro inflammatory cytokine IFNγ is secreted by both the tumor and infiltrating immune cells into the extracellular space acting synergistically to increase HLA expression and enhance antigen presentation (18, 20). Although the effects of IFNγ within the TME have been extensively studied, its effect on the immunopeptidome has only received recent interest (8, 9, 19, 20, 23, 42). HLA silencing is a common tumor mechanism to evade cellular immunity, with tumors containing IFNγ or “inflamed” tumors conferring an improved prognosis and higher overall susceptibility to immunotherapy approaches such as immune checkpoint inhibitors (43). Therefore, it can be speculated that the immunopeptidome of IFNγ exposed tumors confer a higher level of anti-tumor immunity due to the presence of IFNγ-dependent peptide antigens. This is an important consideration for future studies wishing to examine the immunopeptidome of cancer cells.

In this study the TNBC model cell line MDA-MB-231 was comprehensively characterized, with in-depth analysis of the transcriptome, proteome, and immunopeptidome of these cells cultured in the presence or absence of IFNγ. Following qualitative assessment of the immunopeptidome we have identified >51,000 unique peptides in HLA-I and >34,000 unique peptides in HLA-II peptidomes. More importantly we have identified 54,440 previously unreported HLA-I and HLA-II-restricted peptides of which 23,386 were 8–12 mers, adding a valuable source of peptide antigens to the literature. IFNγ treatment caused considerable remolding of the immunopeptidome with typically less than 50% overlap observed between the induced and basal immunopeptidomes. Moreover, an increase in longer peptides (towards 10- and 11-mers) and decreased representation of HLA-A and HLA-C binding peptides was also observed. This change was mirrored at the HLA-B transcript and protein level with allotypes from these loci dominating the immunopeptidome following IFNγ treatment. Consistent with this observation, HLA-B modulation following IFNγ treatment has been highlighted in previous studies (8, 20). However, here we show that these HLA-B binding peptides include a higher proportion of 10- and 11-mer peptides. We speculate that the appearance of these longer peptides within the immunopeptidome are either or a combination of the effects of IFNγ induction at the APPM favoring the generation of longer peptides, or that the increased and abundant expression of nascent HLA-I allotypes facilitates the binding of longer peptide precursors under less competitive binding conditions. The mechanistic basis of these observations will be the subject of future studies.

A key observation made in this study addresses the mechanism of the IFNγ mediated remolding of the immunopeptidome. Here we show a large overlap in the expression of protein antigens between both treatment conditions (93%). However, there is a considerable increase in source protein representation in the HLA-I immunopeptidome (2,343 new source proteins) following cytokine treatment. Moreover, IFNγ-treatment increased the number of distinct peptides derived from a given antigen thereby increasing the coverage of the source protein and enhancing the chances of T cell recognition. One of the reasons this shift occurred may be due to IFNγ increasing the overall expression level HLA-peptide complexes, circumventing some of the challenges in peptide identification and allowing them to be more readily identified *via* mass spectrometry. Furthermore, the shift observed within the peptide repertoire was not proportional to changes within source protein abundance. This highlights that IFNγ induction leads to an enhancement of antigen processing and presentation independent of source protein abundance.

We next examined if previously determined protein degradation rates for some of the source antigens lead to enhanced processing and presentation of these antigens. This analysis also did not provide an explanation for the observed changes in the immunopeptidome following IFNγ stimulation. This is in accordance with a previous study that has suggested HLA peptide presentation is determined by HLA availability rather than peptide supply (20). Consistent with this observation a higher number of potential HLA-B binders entered the immunopeptidome in association with increases in the evidence for these HLA allotypes in the transcriptome and proteome following IFNγ treatment. Furthermore, this trend was not correlated with protein or transcript abundance, supporting the notion that peptide pools exist within the endoplasmic reticulum and these peptides are then bound to available HLA molecules based on their affinities (20).

A higher availability of HLA molecules in the ER promotes increased peptide presentation and this becomes clinically significant as it unveils a suite of antigens which would have been previously missed. This was supported when examining cancer associated antigens, highlighting the increased coverage and the increased diversity of peptides presented with therapeutic relevance. Overall, examining the immunopeptidome in the context of inflammation not only mimics the physiological environment of a tumor more accurately, it also increases the scope and potential to identify more antigens that can be exploited through vaccine approaches. This was particularly evident when comparing tumor associated showing an overall increase in the number and label free intensities of these antigens of interest. Although these peptides have not been tested for their immunogenicity, they are potential sources to be examined in future research. This dataset also encompassed previously reported immunogenic epitopes that were considered to be HLA-A*02:01 restricted and immunogenic based on their annotations in the IEDB (44): with 28 peptides in IFNγ untreated and 32 peptides in IFNγ treated samples. It is important to consider that IFNγ treatment mimics the phenotype of a “hot” tumor, a phenotype associated with improved immunotherapy success (45). Our data also suggest that the enhanced presentation of HLA:B resident peptides could be one of the factors that provide a higher degree of anti-tumor immunity in such “hot” tumors (8).

Taken together these results highlight the plasticity of the TNBC immunopeptidome following IFNγ treatment. Importantly, we show that these changes are not mirrored by intracellular changes in protein and transcript abundance but rather the enhancement of the APPM following cytokine stimulation. The combined dataset from basal and cytokine stimulated MD-MBA-231 TNBC cells represents one of the most comprehensive HLA peptide dataset from a single cell line with over 84,000 linear peptides identified with high confidence. This study provides a comprehensive understanding of the role of cytokine derived changes to the immunopeptidome of TNBC, which will act as a critical consideration for the development of personalized vaccine therapy.

## Data Availability Statement

The datasets presented in this study can be found in online repositories. The names of the repository/repositories and accession number(s) can be found below: PRIDE repository, accession numbers: PXD023038 and PXD023044.

## Author Contributions

GG, NC, PF, and AP conceived and planned all experiments. GG, DD, and RA carried out the experiments. NC, PF, and GG contributed to mass spectrometry analyses. KM, NC, PF, and GG contributed to bioinformatics analyses. GG, NC, PF, and AP wrote the manuscript. All authors contributed to the article and approved the submitted version.

## Funding

This project was funded in part by Australian National Health and Medical Research Council (NHMRC) Project Grants 1165490 (to AP) and 1084283 (to AP and NC). AP is supported by a NHMRC Principal Research Fellowship (1137739). PF is the recipient of a Fellowship from the Victorian Government Department of Health and Human Services acting through the Victorian Cancer Agency.

## Conflict of Interest

The authors declare that the research was conducted in the absence of any commercial or financial relationships that could be construed as a potential conflict of interest.

## Supplementary Material

Supplementary Figure 1 Flow cytometry staining of HLA class I and class II expression following IFNγ stimulation. **(A)** Staining of IFNγ treated cells [orange (50 IU for 48 h)] and without IFNγ treatment (blue) with HLA-I pan antibody; w6/32. The secondary control is labeled in red. **(B)** RM5.112 (pan HLA-class II Ab) staining with IFNγ treated cells [red (50 IU for 48 h)] whilst without IFNγ treatment is marked in yellow which is overlayed on top of the secondary only control which is in green. C-F show median fluorescence intensity (MFI) of HLA-I and HLA-II staining following a time course at 50 IU **(C–D)** and an IFNγ titration for 48 h **(E–F)**. Results are from three biological replicates with mean ± SEM. Statistical testing was performed using a one-way ANOVA using Dunnett’s multiple comparisons test.

Supplementary Figure 2 Overlap between MS based identification of surface HLA-bound peptides from three biological replicates of IFNγ untreated (n = 24,247) and IFNγ treated (n = 46,929). These samples were grouped by their respective condition and compared against each replicate **(A–F)**. **(G)** denotes the complete overlap between all identified HLA-I peptides and their corresponding source proteins for IFNγ untreated is denoted by **(H)** (n = 6,674) (n = 8,565). Comparative analysis of HLA-I and HLA-II source protein and peptide identification are also shown **(I–J)**.

Supplementary Figure 3
**(A)** Bar graph of HLA-II source proteins identified across three biological replicates following IFNγ treatment (mean of 2,753). **(B)** Length analysis of HLA-II peptides derived from MDA-MB-231 cells following IFNγ treatment. **(C)** The proportion of total binders following IFNγ treatment with binding prediction from NetMHCpan 4.0 (mean ± SD) (mean of 80.8 and 90.2 for IFNγ untreated and treated respectively). **(D)** denotes a length analysis following peptides allocated to HLA-A, -B, and -C with **(E–F)** showing each HLA allele separately.

Supplementary Figure 4 Consensus binding motifs via Gibbs Clustering of unique IFNγ treated and untreated 9 mers. **(A)** untreated and IFNγ treated **(B)** MDA-MB-231 cells of unique class I 9 mers only. Figures were generated by Seq2Logo and the contributing peptides to each group are also shown.

Supplementary Figure 5 Analysis of shared source proteins n = 3,327 following IFNγ treatment comparing overall coverage **(A)**, total peptides **(B)**, and overall peptide fold change following treatment **(C)**. Statistical analysis was performed by using a two-way ANOVA and significance is highlighted by **** (P < 0.001). All data are from three biological replicates per condition.

Supplementary Figure 6 Three hundred cancer specific or associated antigens are then plotted to show the overall distribution of HLA peptides originating from both IFNγ treated and untreated or peptides unique to each condition.

Supplementary Figure 7 A volcano plot denoting the transcriptome **(A)** distribution following IFNγ treatment, a line at y = 1.3 is shown to depict differentially expressed transcripts (FDR < 0.05). A comparative analysis between the common identified significant transcripts (FDR < 0.05) and proteins (Q < 0.05) are shown in **(B)** (n = 220). The corresponding log_2_ fold change of these genes and proteins were plotted with a selection of antigen presentation pathway associated genes shown in blue. Correlative analysis following a Pearson correlation test between 220 transcripts and proteins revealed a 95% CI of 0.66 and 0.78 with a correlation of 0.73. The p value reported was 3.37e-37. A volcano plot denoting the proteome changes following IFNγ treatment is shown in **(C)** with a line at y = 1.3 to denote differentially expressed proteins. The overlap of all identified transcripts and proteins are shown in **(D)**.

Supplementary Figure 8 Scatter plot between number of HLA-I peptides following untreated **(A)** and IFNγ treated **(B)** samples with corresponding half-life data from Lamond et al. A Pearson correlation was conducted showing a Pearson correlation of coefficient of −0.003 **(A)** and −0.02 **(B)**, both of these results yielded an insignificant p value 0.8 and 0.15 (A and B respectively). The top 100 half-lives considered to be the Rapidly degrading proteins (RDP) were analyzed with the number of HLA-I peptides from untreated **(C)** and IFNγ treated **(D)**. Following a Pearson correlation test an insignificant p value was determined (0.19 untreated and 0.47 for IFNγ treated) with a correlation coefficient of 0.131 and 0.072 respectively. Furthermore, the top 100 most stable half-lives were also analyzed with the number of HLA-I peptides from untreated **(E)** and IFNγ treated **(F)**. Following a Pearson correlation test an insignificant p value was determined (0.81 untreated and 0.85 for IFNγ treated) with a correlation coefficient of 0.025 and 0.019 respectively.

Supplementary File Table of cancer antigens.
