# Supplementary material for: IFNγ Modulates the Immunopeptidome of Triple Negative Breast Cancer Cells by Enhancing and Diversifying Antigen Processing and Presentation
Source: Front Immunol. 2021 Apr 22;12:645770. doi: 10.3389/fimmu.2021.645770 (PMC8100505; doi:10.3389/fimmu.2021.645770)
Supplement: Supplementary Figure 1 — Flow cytometry staining of HLA class I and class II expression following IFNγ stimulation. (A) Staining of IFNγ treated cells [orange (50 IU for 48 h)] and without IFNγ treatment (blue) with HLA-I pan antibody; w6/32. The secondary control is labeled in red. (B) RM5.112 (pan HLA-class II Ab) staining with IFNγ treated cells [red (50 IU for 48 h)] whilst without IFNγ treatment is marked in yellow which is overlayed on top of the secondary only control which is in green. C-F show median fluorescence intensity (MFI) of HLA-I and HLA-II staining following a time course at 50 IU (C–D) and an IFNγ titration for 48 h (E–F). Results are from three biological replicates with mean ± SEM. Statistical testing was performed using a one-way ANOVA using Dunnett’s multiple comparisons test. [file Presentation_1.pptx]

## Slide 1
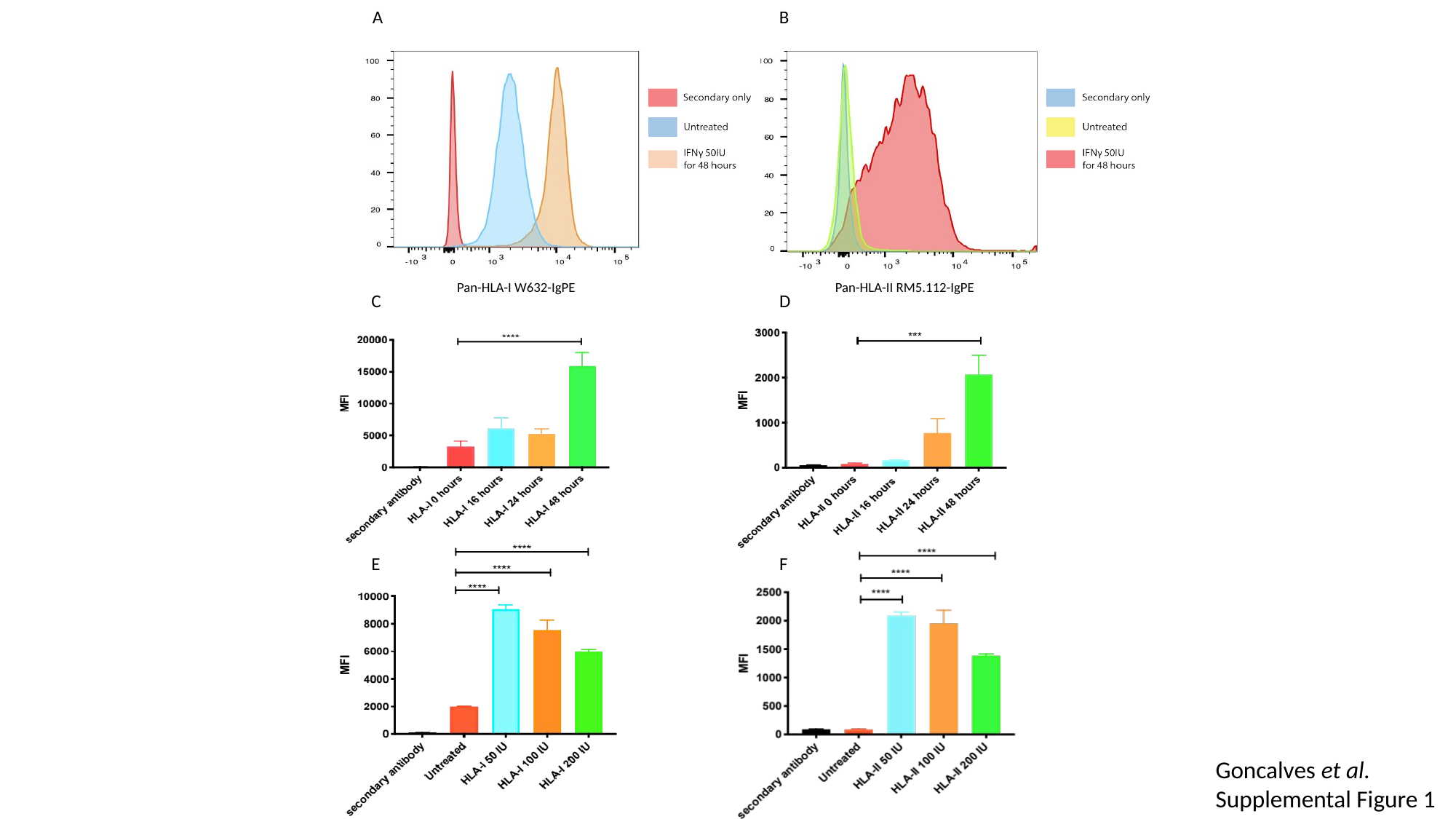

A
B
Pan-HLA-I W632-IgPE
Pan-HLA-II RM5.112-IgPE
C
D
E
F
Goncalves et al. Supplemental Figure 1

## Slide 2
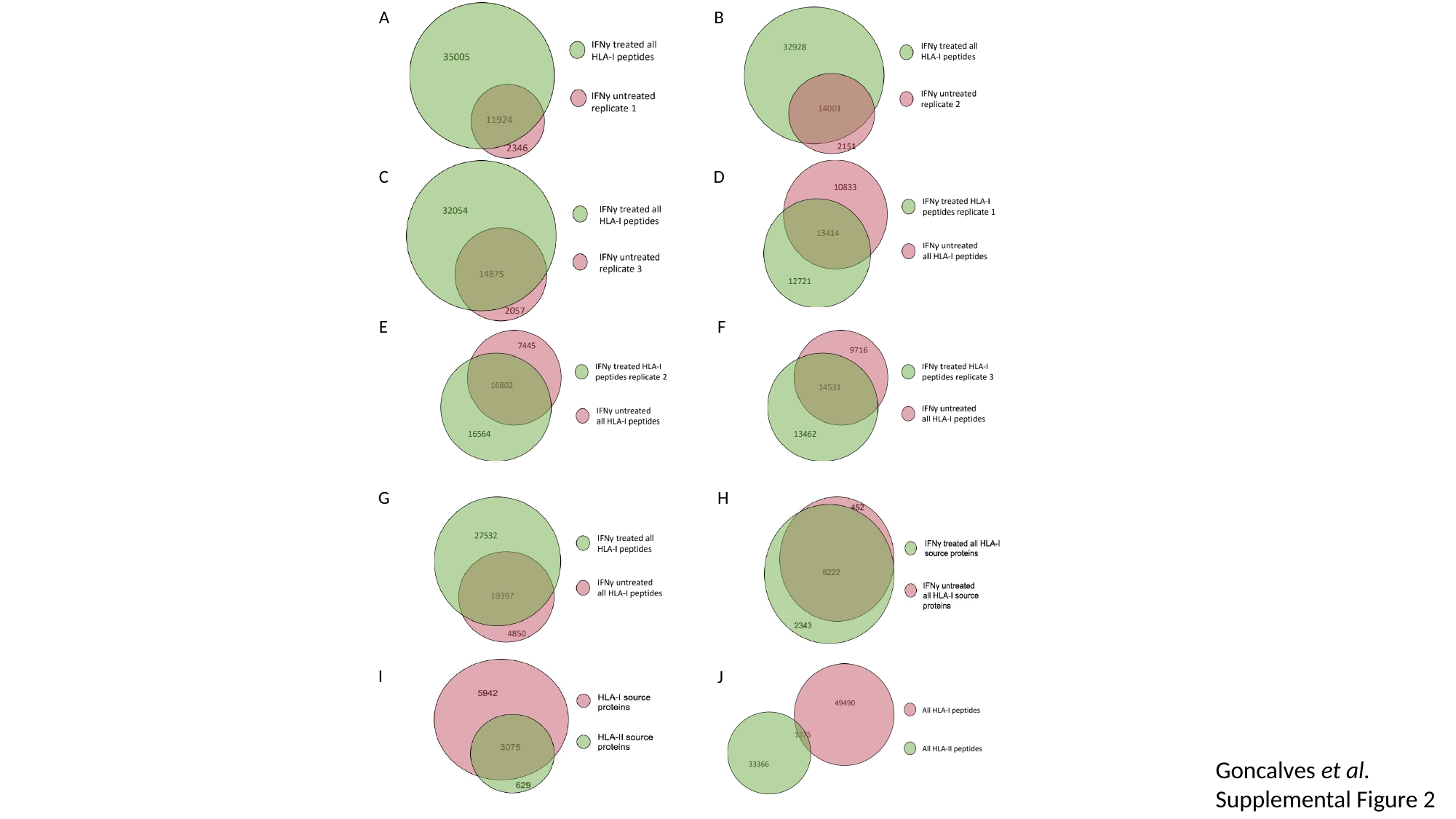

B
A
D
C
F
E
G
H
I
J
Goncalves et al. Supplemental Figure 2

## Slide 3
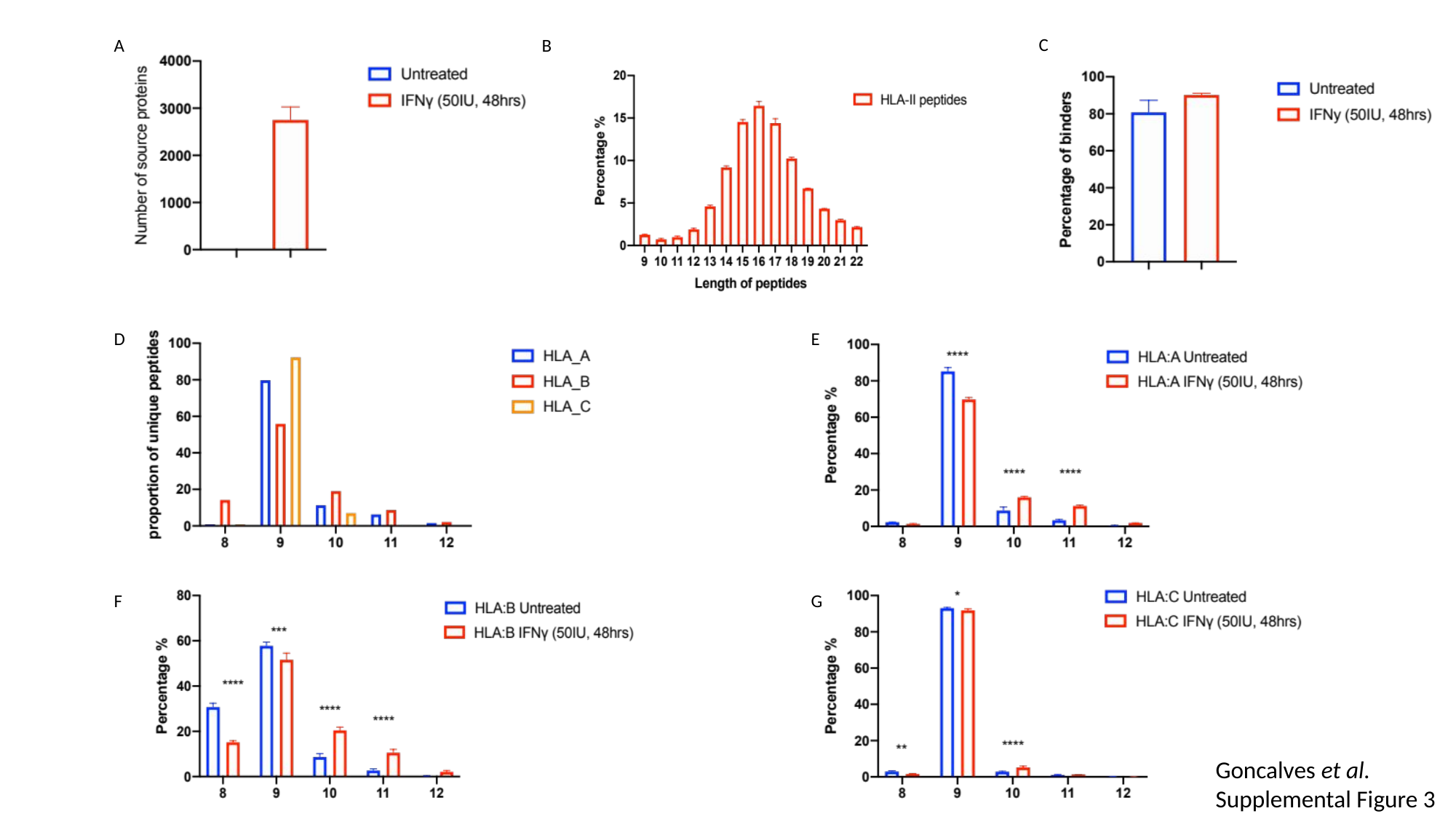

C
A
B
D
E
F
G
Goncalves et al. Supplemental Figure 3

## Slide 4
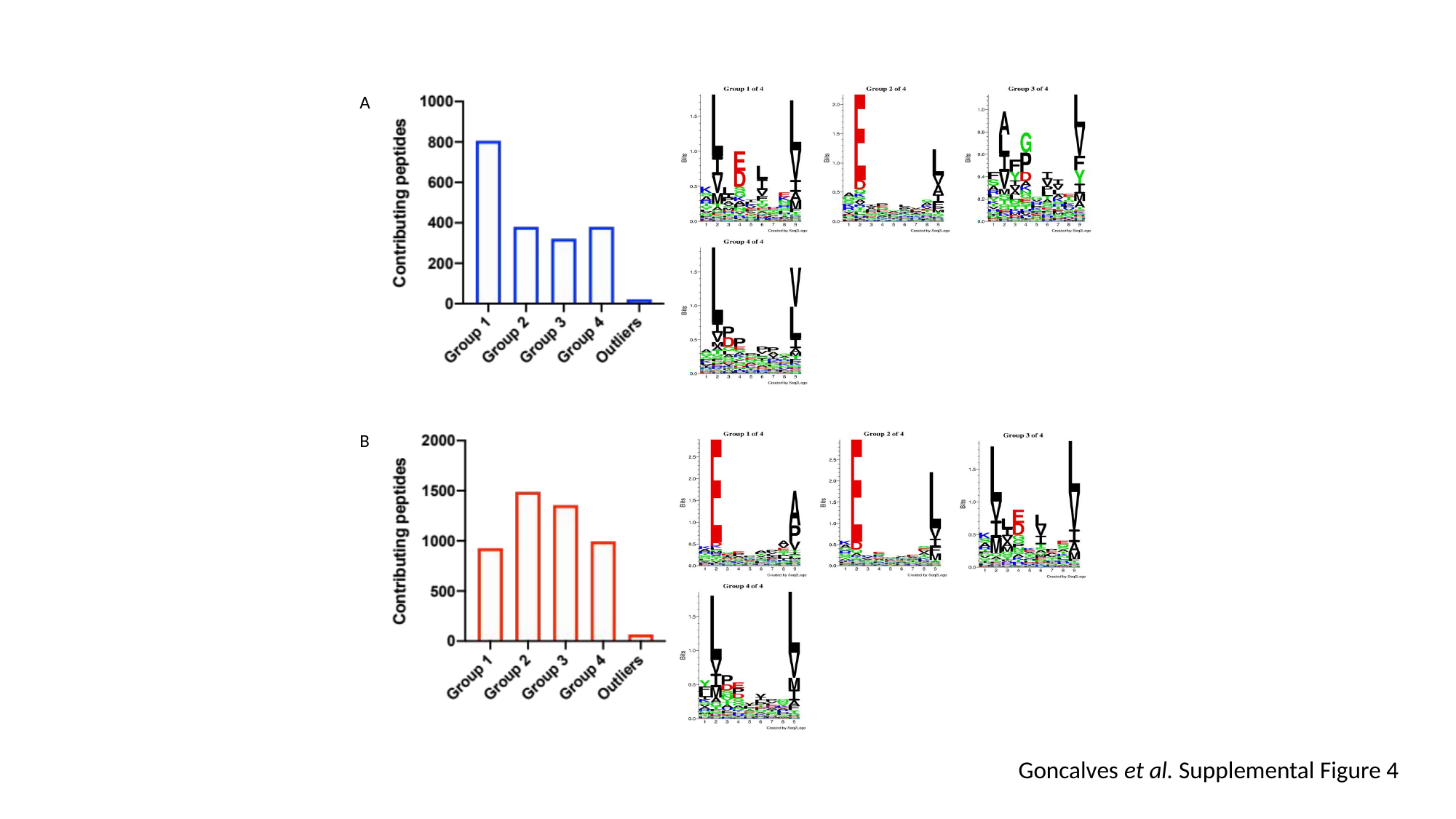

A
B
Goncalves et al. Supplemental Figure 4

## Slide 5
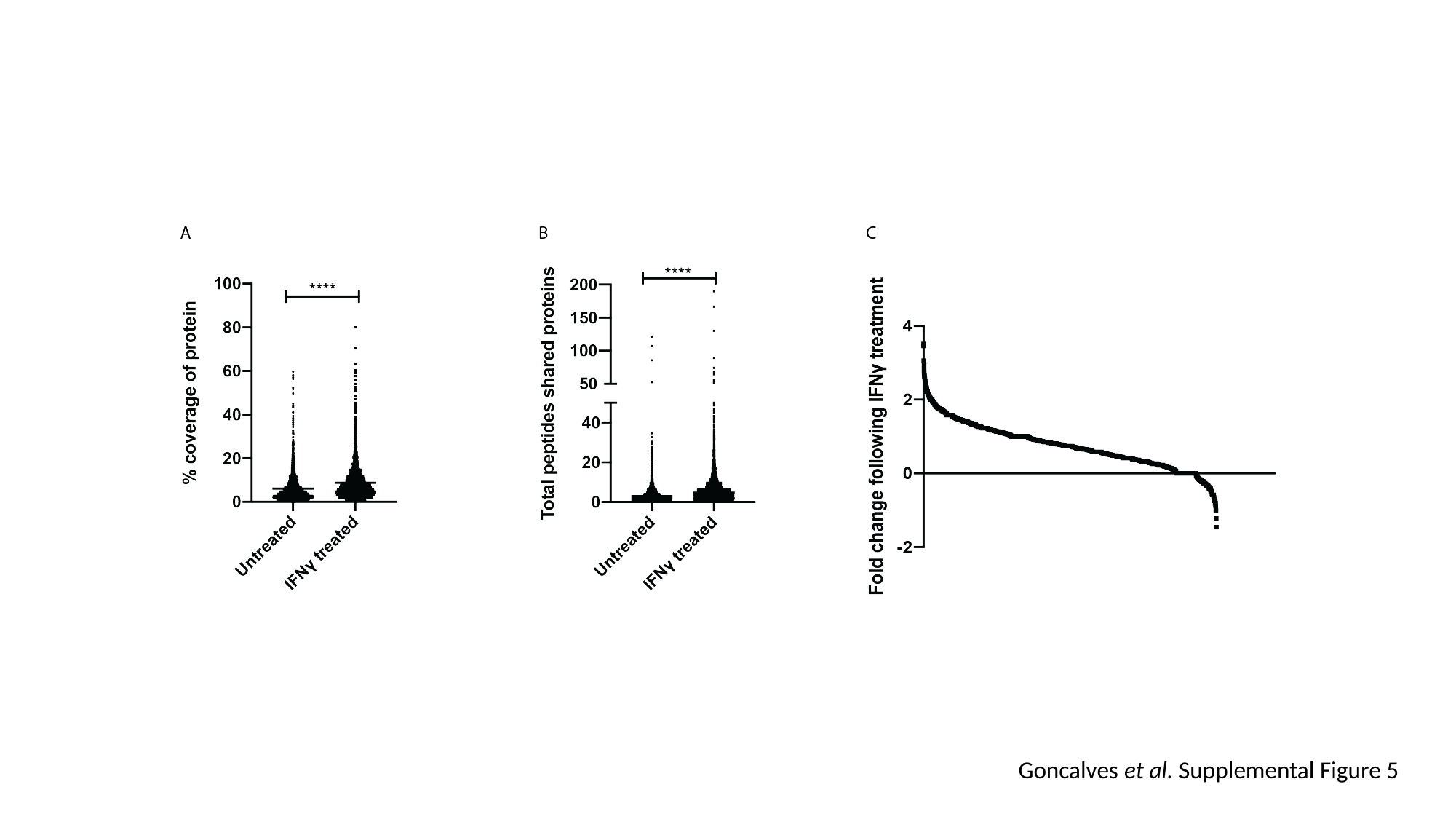

Goncalves et al. Supplemental Figure 5

## Slide 6
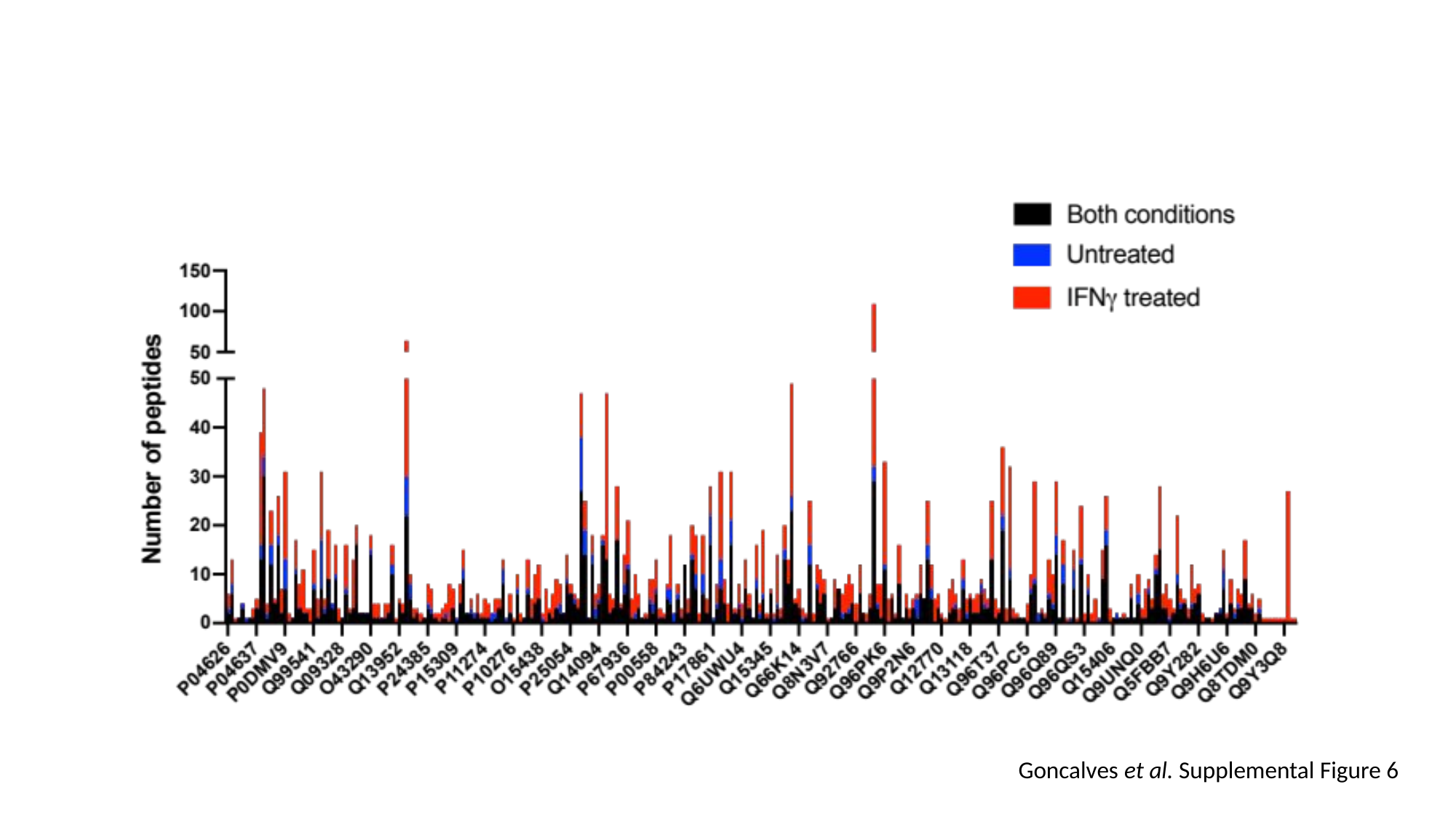

Goncalves et al. Supplemental Figure 6

## Slide 7
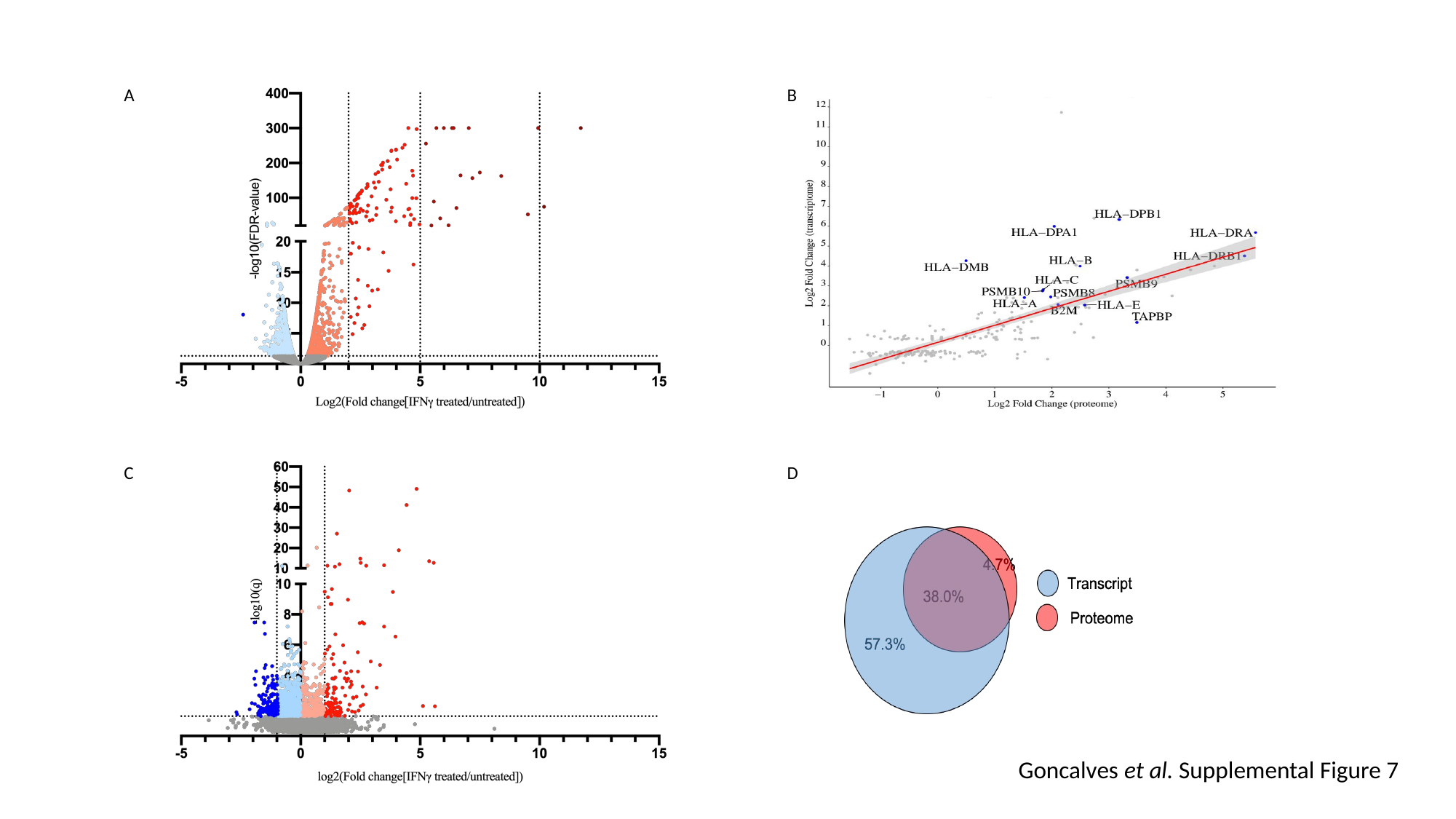

A
B
C
D
Goncalves et al. Supplemental Figure 7

## Slide 8
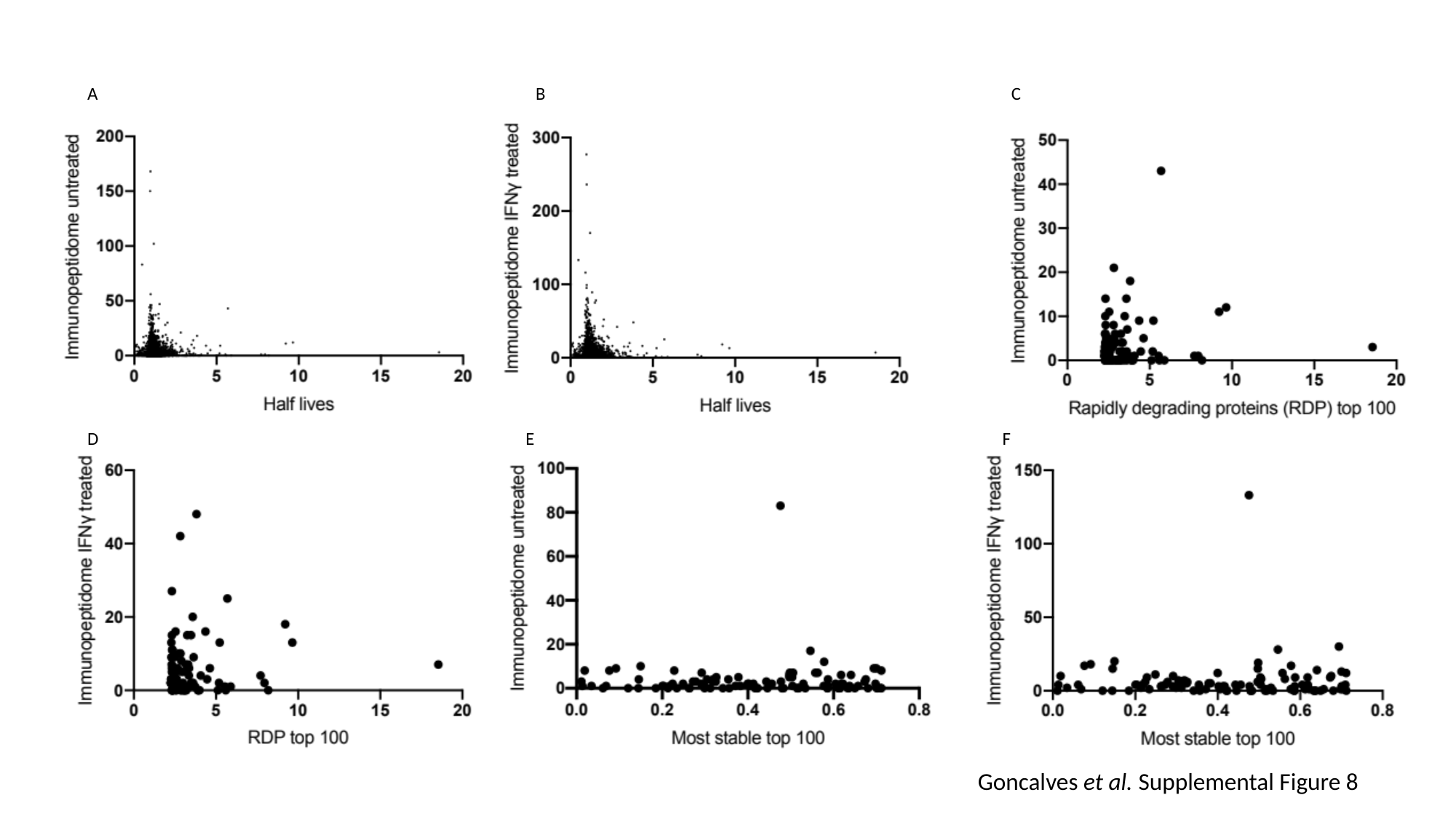

C
B
A
F
D
E
Goncalves et al. Supplemental Figure 8
